# Crimean-Congo Hemorrhagic Fever Virus in Cattle and Ticks, Israel

**DOI:** 10.3201/eid3111.250622

**Published:** 2025-11

**Authors:** Nir Rudoler, Marisol Rubinstein-Guini, Asael Roth, Victoria Indenbaum, Oran Erster, Yaniv Lustig, Elad Eliahoo

**Affiliations:** Ministry of Agriculture and Food Security, Beit Dagan, Israel (N. Rudoler, M. Rubinstein-Guini, A. Roth, E. Eliahoo); Ministry of Health, Ramat-Gan, Israel (V. Indenbaum, O. Erster, Y. Lustig); Sheba Medical Center, Ramat-Gan (V. Indenbaum, O. Erster, Y. Lustig); Tel Aviv University Faculty of Medical and Health Sciences, Tel Aviv, Israel (Y. Lustig)

**Keywords:** Crimean-Congo hemorrhagic fever virus, CCHFV, tickborne disease, zoonoses, hemorrhagic fever, ixodid, viruses, vector-borne infections, Israel

## Abstract

We conducted a nationwide serologic and molecular survey to elucidate the epidemiologic status of Crimean-Congo hemorrhagic fever virus in Israel. We found serologic and molecular evidence of virus circulation in the country. Future human cases could be prevented by increasing public awareness and implementing public health measures.

Crimean-Congo hemorrhagic fever virus (CCHFV) is an enveloped segmented negative-sense RNA virus belonging to the *Nairoviridae* family of the *Bunyavirales* order (*1*). The virus is the etiologic agent of Crimean-Congo hemorrhagic fever (CCHF), a severe tickborne zoonotic illness with a wide geographic distribution, infecting ≈10,000–15,000 humans annually worldwide (*2*). Ticks, primarily of the genus *Hyalomma*, are considered the vector and the reservoir of CCHFV (*3*). CCHFV can infect various animal hosts, including livestock, that, although remaining asymptomatic, can act as amplifying hosts of the virus (*4*). CCHFV is transmitted to humans primarily through the bite of an infected tick but also by direct contact with blood or body fluids of infected animals or humans or through improperly sterilized medical equipment (*1*).

Neither CCHFV infection in humans nor positive serologic test results in humans or in animals were previously reported in Israel. However, outbreaks of the disease and seropositivity among livestock have been reported in neighboring countries (*5*). Moreover, the main vector of CCHFV, the *Hyalomma marginatum* tick, is endemic in Israel (*6*). Hence, the risk for CCHFV emergence in Israel is considered high, and undetected viral circulation might already exist in specific regions of the country (*6*).

During April 2024–February 2025, we sampled whole blood, serum specimens, and ticks from 19 beef cattle herds from different regions in Israel. We tested serum samples by using an ELISA commercial kit (ID Screen CCHF Double Antigen Multi-species; Innovative Diagnostics, https://www.innovative-diagnostics.com). We classified the ticks morphologically and extracted RNA to identify CCHFV presence (Appendix).

Sixteen beef cattle herds had serologic evidence of prior exposure to CCHFV; seropositivity ranged from 3% to 100% (Table). We detected virus exposure eliciting an immune response in locations across Israel (Figure). Those serologic results are comparable with rates reported in CCHFV-endemic countries with confirmed human cases, such as Turkey (Türkiye) (*7*) and Pakistan (*8*). Consistent with prior publications linking older age with seropositivity (*9*), the age of the sampled animals from the 2 herds that were seronegative was <2 years. Moreover, although all serum samples from the heifers (<2 years of age) of the Keshet herd (Golan Heights) were seronegative, subsequent sampling of older cows (3–15 years of age) in the herd revealed 100% positive serologic test results (Table). We also tested serum samples that were randomly collected from 200 wild animals dispersed through the country (including boars, foxes, jackals, ibexes, dogs, deer, oryxes, gazelles, and porcupines) during 2023–2024. We found that only 2 samples, from ibexes (*Capra nubiana*) residing in the Negev Desert (southern Israel), were seropositive for CCHFV antibodies (Figure).

**Table:** Seroprevalence of Crimean-Congo hemorrhagic fever virus in cattle, Israel, April 2024–February 2025

Site of sampling (district)	No. sampled animals	No. (%) seropositive	Average age of sampled animals, y
Kidmat Tzvi (Golan Heights)	34	17 (50)	8.3
Merom Golan (Golan Heights)	39	14 (35.9)	9.3
Ramat Magshimim (Golan Heights)	39	6 (15.4)	8.6
Keshet (Golan Heights)*	30	0	<2
Keshet (Golan Heights)	20	20 (100)	8.08
Ortal (Golan Heights)	25	22 (88)	8.8
Gazit (Yizrael Valley)	30	9 (30)	7.7
Zipori (Yizrael Valley)	17	5 (29.4)	7.05
Alonim (Yizrael Valley)	31	1 (3.2)	2^†^
Na’aura (Gilboa)	21	6 (28.6)	7.5
Nurit (Gilboa)	22	1 (4.7)	5^†^
Gal’ed (Ramat Menashe)	33	10 (30.3)	5.8
Ein Yaakov (Western Galilee)	24	19 (79.1)	8.5
Shetula (Western Galilee)	24	2 (8.3)	2.5
Lapidot (Western Galilee)	30	2 (6.6)	11.8
Kochav Hayarden (Jordan Valley)*	17	0	<2
Kfar Szold (Hula Valley)	30	1 (3.3)	3^†^
Sha’alabim (Gezer)	32	27 (84)	5.7
Avi’ezer (Adulam)*	9	0	<2
Binyamina (Haifa)	29	19 (65.5)	6.07
Total no. samples	536	181 (34)	

*Heifers only. †Estimated average age provided by owner.

**Figure:**
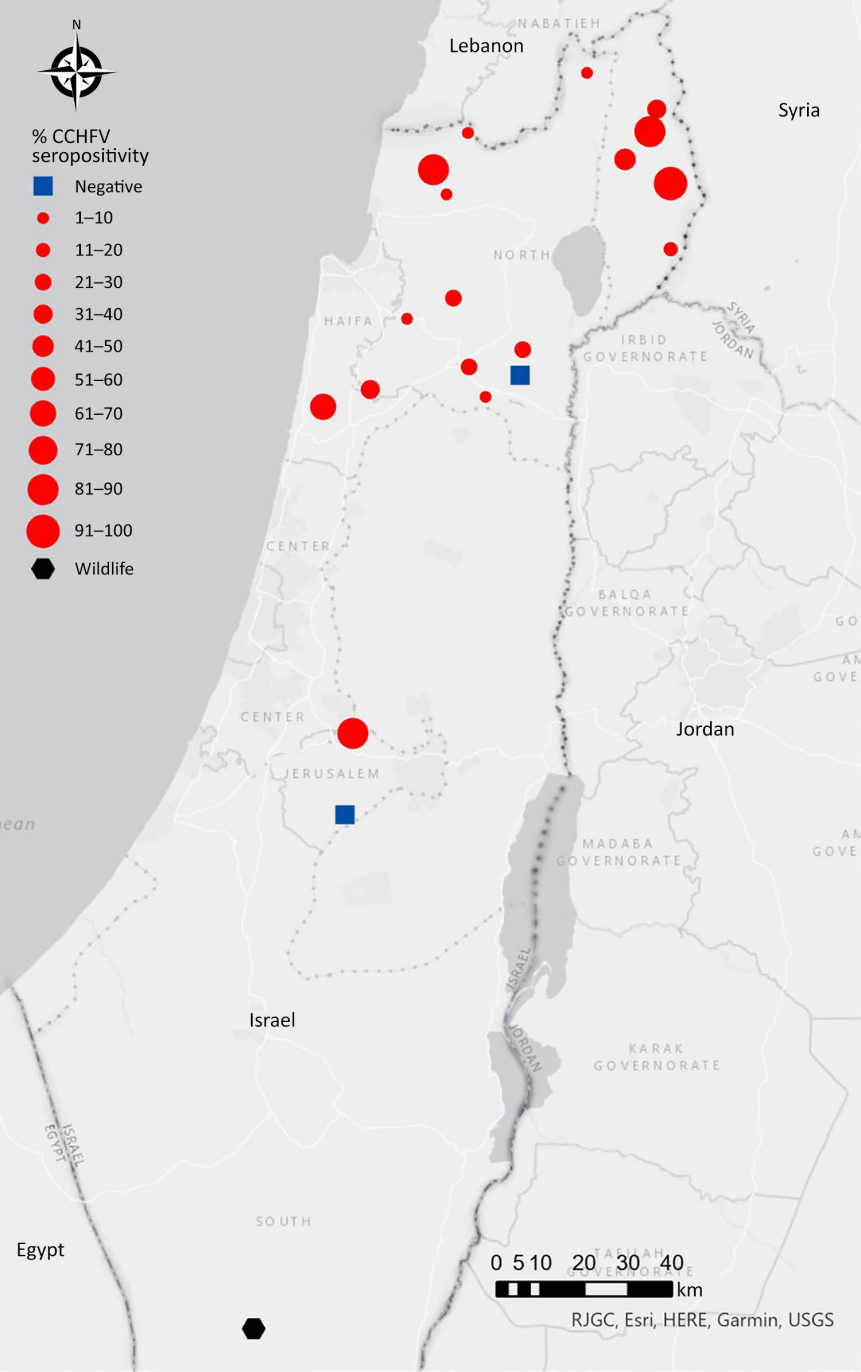
Distribution of samples seropositive for CCHFV in study of serologic and molecular evidence of CCHFV in cattle and ticks, Israel, April 2024–February 2025. Red dots represent seropositive beef cattle herds. Size of dots correlates with percentage of CCHFV seropositivity. Blue rectangles represent CCHFV seronegative herds. Black hexamer represents 2 of 200 CCHFV seropositive wild animals that were tested. Map created by using ArcGIS software (Esri, https://www.esri.com). CCHFV, Crimean-Congo hemorrhagic fever virus.

We tested for CCHFV RNA in 227 ticks retrieved from 8 cattle herds and 51 ticks retrieved from wildlife by using quantitative reverse transcription PCR targeting 2 regions of the small segment (*10*). Because we tested each tick individually, we determined a sample to be positive only when both regions were amplified. In addition, the positive samples were validated at an independent facility (The Central Virology Laboratory, Ministry of Health and Sheba Medical Center, Ramat-Gan, Israel). Of the 227 ticks from cattle, 23 (10%) samples collected from northern Israel (Golan Heights and Western Galilee) were positive for CCHFV (Appendix Table 1). Likewise, among 51 ticks collected from wild animals (all sampled from northern Israel), 7 (13%) were positive for CCHFV (Appendix Table 2). All ticks positive for CCHFV belonged to 2 genera, *Hyalomma* and *Rhipicephalus* (Appendix Tables 1, 2). Sanger sequencing of the 181-bp (1,068–1,248 nucleotides at the small segment) amplicons of 10 samples, which we successfully amplified by using endpoint reverse transcription PCR (*10*), followed by phylogenetic analysis, indicated that the sequences clustered to the Asia-1 genotype (Appendix Figure).

We tested serum samples from 13 persons who had close contact with either CCHFV-positive ticks or seropositive cattle for the presence of CCHF IgG by using ELISA (Euroimmun, https://www.euroimmun.com). All persons tested were seronegative. The study was conducted with the approval of the Shiba Medical Center institutional review board (approval no. 1601–24-SMC).

We present evidence of CCHFV circulation in Israel, expanding the known geographic distribution of CCHFV in the Middle East. We have found serologic evidence of prior exposure to CCHFV in livestock and in wild animals; we also detected CCHFV in the ticks infesting them. Furthermore, the seroprevalence in the cows was found to be comparable to seroprevalence in CCHFV-endemic countries with proven human cases. In addition, the seropositivity prevalence in the cattle and the observation that cows <2 years of age were not seropositive suggest that the virus has been circulating for several years. Still, the route of introduction remains unclear. Our results highlight the importance of raising public and clinical awareness of CCHF, especially among high-risk populations, despite the current absence of human cases.

Appendix Additional information about evidence of Crimean-Congo hemorrhagic fever virus in cattle and ticks, Israel.
