# Supplementary material for: Crimean-Congo Hemorrhagic Fever Virus in Cattle and Ticks, Israel
Source: Emerg Infect Dis. 2025 Nov;31(11):2182–5. doi: 10.3201/eid3111.250622 (PMC12704528; doi:10.3201/eid3111.250622)
Supplement: Appendix — Additional information about evidence of Crimean-Congo hemorrhagic fever virus in cattle and ticks, Israel. [file 25-0622-Techapp-s1.pdf]

Article DOI: https://doi.org/10.3201/eid3110.250622
EID cannot ensure accessibility for supplementary materials supplied by authors. Readers who have difficulty accessing supplementary content should contact the authors for assistance.

Evidence of Crimean-Congo Hemorrhagic Fever Virus in Cattle and Ticks, Israel

Appendix

Materials and Methods

Sample collection

A total of 19 herds were sampled in several regions, spanning from the north to central Israel, to detect Crimean-Congo hemorrhagic fever virus (CCHFV). The cows were sampled for serum, total blood, and ticks. Three herds were composed of heifers (≤2 years old). The average age of the cows in the other 16 herds was 7 years (range, 3–21 years). The average number of samples from each herd was 27 (range, 9–39).

Ticks

After collection, the ticks were kept dry in –80 °C until they were classified visually. Identification of ixodid ticks species was performed as described before (*1*). For disruption and RNA extraction, the ticks were manually sliced at their longitudinal axis. Next, the ticks were incubated with 250 µl of lysis buffer (IndMag Pathogen IM48 Cartridge, Indical bioscience) supplemented with proteinase K (100 µg/ml) for 3 hours at 56°C. Then, the samples were vortex for 1 minute followed by spinning down. Lastly, 200 µl of the supernatant of each sample was transferred to the cartridge for RNA extraction by IndiMag 48s instrument (Indical bioscience).

ELISA

Thirty microliters of serum were subjected to testing in duplicates by using multispecies double antigen CCHFV antibodies commercial ELISA plate (ID Screen, CCHF Double Antigen Multi-Species, REF CCHFDA-5P, Innovative Diagnostics) as detailed in the manual. Next, the optical density (OD) in 450 nm was measured using Synergy H1 microplate reader (BioTek). Validation of the test was calculated as ODpc >0.350 and ODpc/ODnc >3. Sample's reactivity

was calculated as percentage of the positive control reactivity (S/P%) where positive serum threshold is S/P% >30%.

Real-time reverse transcription PCR

Real-time reverse transcription PCR (RT-PCR) was performed by using 2 separated systems targeting the S segment as previously described (*2*,*3*). Both systems were used in an assay with the following protocol: 20 µL reaction volume, 5 µL RNA and 1× concentration of buffer and enzymes from the qScript XLT One-Step RT-qPCR (QuantaBio) by using QuantStudio5 real-time PCR system (Applied Biosystem, Thermo Fisher Scientific). For the first system, we used 600 nM of the forward primer (designated RWCF, CAAGGGGTACCAAGAAAATGAAGAAGGC), and 600 nM of the reverse primer (designated RWCR, GCCACAGGGATTGTTCCAAAGCAGAC). In addition, we used 3 probes in concentration of 100 nM each. The sequences of the probes were as followed: SE01 (FAM-ATCTACATGCACCCTGCTGTGTTGACA-TAMRA -broad range probe), SE03 (FAM-ATTTACATGCACCCTGCCGTGCTTACA-TAMRA) and SEoA (FAM-AGCTTCTTCCCCCACTTCATTGGAGT-TAMRA) (*2*). The cycling conditions used were 50 °C for 15 min, 95 °C for 15 min, followed by 45 cycles of 94 °C for 15 sec and 59 °C for 30 sec (with quantification analysis of fluorescence performed at the end of each 59 °C step). For the second system, we used 900 nM of the forward primer (designated CCHF S1, TCT CAA AGA AAC ACG TGC C), and 900 nM the reverse primer (designated CCHF S122, CCT TTT TGA ACT CTT CAA ACC) and 625 nM of the probe (designated CCHF probe, FAM-ACT CAA GGK AAC ACT GTG GGC GTA AG-BHQ1) (*3*). The cycling conditions used were 50 °C for 10 min, 95 °C for 2 min, followed by 45 cycles of 95 °C for 10 sec and 60 °C for 40 sec (with quantification analysis of fluorescence performed at the end of each 60 °C step) and a final cooling step of 40 °C for 30 sec.

RT PCR

End point RT-PCR was conducted to sequence partial parts of CCHFV genome. First, cDNA was synthesized by using SensiFAST cDNA Synthesis Kit (Meridian Bioscience), which includes a unique blend of random hexamers and anchored oligo (dT) primers. The cycler conditions were 25 °C for 10 min, 42 °C for 15 min and final step of 48 °C for 15 min. Next, amplification of the S segment at index 1,068–1,248 (181bp) was conducted by using repliQa HiFi ToughMix (QuantBio) with addition of 300 nM of primers RWCF and RWCR and 2.5 µl of

the cDNA. The cycling conditions used were 98 °C for 10 seconds, followed by 35 cycles of 98 °C for 10 seconds and 68 °C for 5 seconds (*3*). The products were ran on 1.2% Agarose gel (in 0.5XTAE buffer) and detected by using GelDoc EZ imager (BioRad).

Sanger sequencing

PCR products in the appropriate size (181bp) were purified by using Expin Combo GP mini (GeneAll). The DNA concentration was measured using NanoDrop2000 Spectrophotometers (Thermo Fisher Scientific). Next, the DNA was sent to HyLabs company (Israel) for Sanger sequencing using RWCF or RWCR primers. Finally, the forward and reverses sequencing were aligned to assemble the contigs of the PCR segment sequences.

Phylogenetic analysis

The contigs of the PCR segments obtained in this study were compared with reference S segment sequences of CCHFV strains of different genetic lineages available in GenBank (Appendix Table 3). The phylogenetic analysis of partial S segment sequences was conducted via the neighbor-joining method according to the Tamura-Nei matrix by using Geneious Prime software (GraphPad Software, LLC).

References

<jrn>1. Estrada-Peña A, D'Amico G, Palomar AM, Dupraz M, Fonville M, Heylen D, et al. A comparative test of ixodid tick identification by a network of European researchers. Ticks Tick Borne Dis. 2017;8:540–6. PubMed https://doi.org/10.1016/j.ttbdis.2017.03.001</jrn>

<jrn>2. Wölfel R, Paweska JT, Petersen N, Grobbelaar AA, Leman PA, Hewson R, et al. Virus detection and monitoring of viral load in Crimean-Congo hemorrhagic fever virus patients. Emerg Infect Dis. 2007;13:1097–100. PubMed https://doi.org/10.3201/eid1307.070068</jrn>

<jrn>3. Atkinson B, Chamberlain J, Logue CH, Cook N, Bruce C, Dowall SD, et al. Development of a real-time RT-PCR assay for the detection of Crimean-Congo hemorrhagic fever virus. Vector Borne Zoonotic Dis. 2012;12:786–93. PubMed https://doi.org/10.1089/vbz.2011.0770</jrn>

Appendix Table 1. Prevalence of Crimean-Congo hemorrhagic fever virus–positive ticks in cattle herds

	No. ticks				
		CCHFV positive (%)			
Site of sampling (district)	Total	RT-qPCR 1*	RT-qPCR 2†	RT-qPCR overlap	Tick species
Kidmat Tzvi (Golan Heights)	26	9 (34.6)	4 (15.4)	4 (15.4)	*Hyalomma marginatum, Rhipicephalus turanicus, Rhipicephalus sanguineus.*
Merom Golan (Golan Heights)	74	16 (21.6)	6 (8)	6 (8)	*Hyalomma marginatum, Rhipicephalus turanicus, Rhipicephalus sanguineus, Rhipicephalus excavatum.*
Ramat Magshimim (Golan Heights)	55	19 (34.5)	11 (20)	11 (20)	*Hyalomma marginatum, Rhipicephalus turanicus, Rhipicephalus sanguineus, Rhipicephalus annulatus.*
Keshet (Golan Heights)	25	3 (12)	2 (8)	2 (8)	*Rhipicephalus sanguineus*
Gazit (Yizrael valley)	9	0	0	0	
Binyamina (Haifa)	9	0	0	0	
Lapidot (Western Galilee)	29	1 (3.4)	0	0	*Hyalomma marginatum*
Total samples	227	47 (20.7)	23 (10)	23 (10)	

*Adopted from Wolfel et al (2).
‡Adopted from Atkinson et al (3).

Appendix Table 2. Crimean-Congo hemorrhagic fever virus–positive ticks in wildlife

Site of sampling (district)	Animal	Tick species
Ma’ale Gilboa (Gilboa)	Boar	*Rhipicephalus turanicus*
Mesilot (Gilboa)	Jackal	*Rhipicephalus turanicus*
Ma’ale Gilboa (Gilboa)	Boar	*Rhipicephalus turanicus*
Hamat-Gader (Yarmuk River valley)	Fox	*Rhipicephalus sanguineus*
Amikam (Alona)	Boar	*Rhipicephalus turanicus*
Ramat-Tzvi (Gilboa)	Boar	*Rhipicephalus turanicus*
Mitzpe-Netofa (Lower Galilee)	Boar	*Hyalomma spp.*

Appendix Table 3. Reference nucleotide sequences of Crimean-Congo hemorrhagic fever virus S segment retrieved from the GenBank database

Strain	Country	Year	Genetic lineage	GenBank accession no.
ArD8194	Senegal	1969	Africa-1	DQ211639
ArD15786	Senegal	1972	Africa-1	DQ211640
Congo3010	Congo Republic	1956	Africa-2	DQ144418
CCHFV_Semunya	Uganda	1967	Africa-2	PP735370
SPU94_85_813055_S	South Africa	1985	Africa-2	MF511221
ArD39554	Mauritania	1984	Africa-3	DQ211641
Sudan-AB1–2009	Sudan	2009	Africa-3	HQ378179
SPU34_87_813049_S	Namibia	1987	Africa-3	MF511218
18	Egypt	2020	Africa-3	MW467898
201643792	Spain	2016	Africa-3	MF287636
SPU 44/08	South Africa	2008	Africa-3	KJ682824
Matin	Pakistan	1976	Asia-1	AF527810
CCHF/NIHPAK-31/2023	Pakistan	2023	Asia-1	OR964910
CCHF-2011–49-NIH-PAK	Pakistan	2011	Asia-2	MN970069
CCHF/NIHPAK-37/2024	Pakistan: Punjab	2024	Asia-2	PQ523721
C-68031	China	1968	Asia-2	DQ211642
79121 M18	China	2004	Asia-2	GU477494
AP92	Greece	1975	Europe-2	DQ211638
Pentalofos-Greece-2015	Greece	2015	Europe-2	MG516211
Iran-Gilan69	Iran	2012	Europe-1 (Va subgroup)	KJ0275221
STV/HU29223	Russia, Stavropol territory	2000	Europe-1 (Va subgroup)	AF481802
ROS/HUVLV-100	Russia, Rostov region	2003	Europe-1 (Vb subgroup)	DQ206447
ROS/TI28044	Russia, Rostov region	2000	Europe-1 (Vb subgroup)	AY277672
Kashmanov	Russia, Rostov region	1967	Europe-1 (Vb subgroup)	DQ211644
Yozgat19–2012	Turkey	2012	Europe-1 (Vc subgroup)	KR092375
K229–243	Russia, Astrakhan region	1984	Europe-1 (Vc subgroup)	KX013467
1-CRIMEA/HU-2015	Russia, Crimea Republic	2015	Europe-1 (Vd subgroup)	KU161586
Kosova-Hoti	Kosovo	2001	Europe-1	DQ133507
V46/13	Bulgaria	2013	Europe-1	KR011837
Iran-Gilan69	Iran	2012	Europe-1	KJ027521
CCHFV_Kosovo_2009	Kosovo	2009	Europe-1	PP735364
Tur_2004_813048_S	Turkey	2004	Europe-1	MF511207
CCHFV_1	Turkey (Türkiye)	2020	Europe-1	PP735307

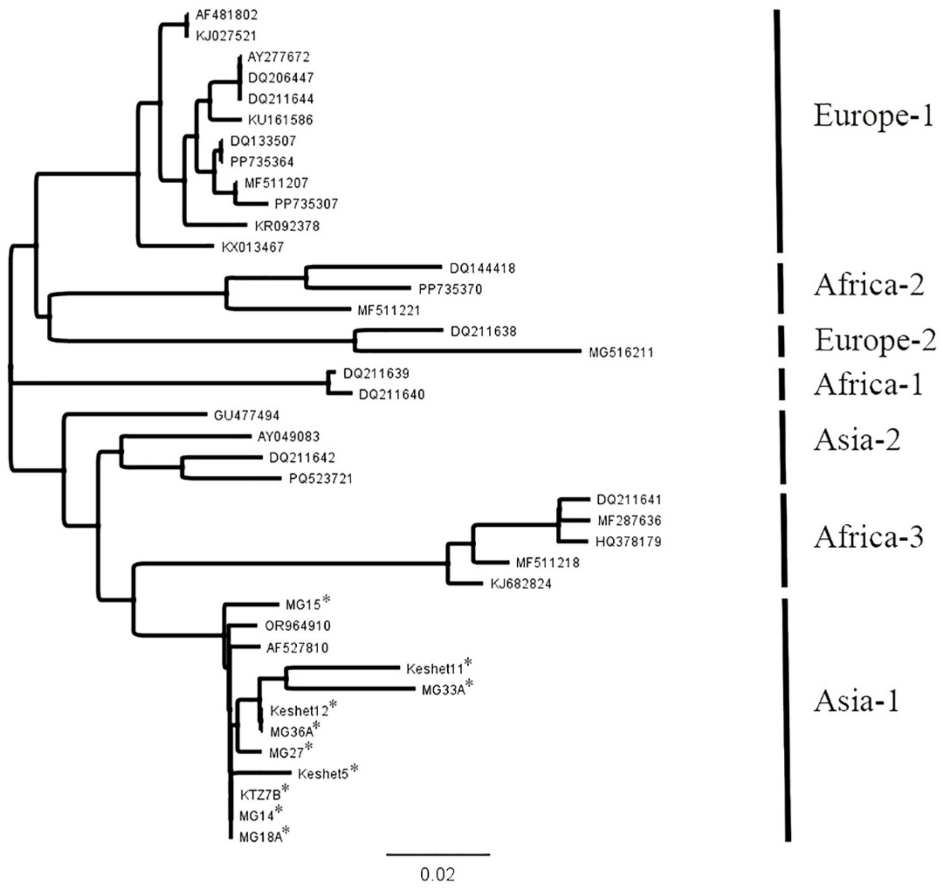

Appendix Figure. Phylogenetic tree among distinct Crimean-Congo hemorrhagic fever virus (CCHFV) isolates based on 181 bases region of the S segment. Thirty distinct CCHFV S segment sequences were retrieved from Genbank to represent selected sequences of the defined 7 CCHFV genotypes from different regions (see Appendix Table 3). Ten sequences (marked with *) from identified positive ticks collected from 3 beef herds (Keshet, Merom Golan, and Kidmat Tzvi) were sequenced. The phylogenetic relationship of all 40 sequences was built by Neighbor-Joining method using Tamura-Nei genetic distance model (Geneious Prime software). Genotypes clusters are indicated for each group. Scale of 0.02 nt substitutions per site.
